# Book Review: Epigenetics in Plants of Agronomic Importance: Fundamentals and Applications

**DOI:** 10.3389/fpls.2019.00882

**Published:** 2019-07-09

**Authors:** Denisa Tomkova, Alexandre Berr

**Affiliations:** Institut de Biologie Moléculaire des Plantes UPR2357 du CNRS, Associé à l'Université de Strasbourg, Strasbourg, France

**Keywords:** epigenetics, plant of agronomic importance, environmental changes, stress response, inheritance

## Introduction

The involvement of epigenetic mechanisms in modulating plant development and stress response is now well-established. Therefore, there is a real and clear need to critically analyse and synthesize the abundant and purportedly relevant information into a single work. This is precisely the purpose of the new version of the book Epigenetics in Plants of Agronomic Importance: Fundamentals and Applications (Alvarez-Venegas et al., 2019). Besides providing a general overview of epigenetic mechanisms in model plants and in crops of economical relevance, authors critically highlight recent insights into the involvement of epigenetic mechanisms in the control of plant developmental processes, especially in shaping phenotypic plasticity to any kind of environmental changes. Authors even went a step further by emphasizing the importance of such knowledge and their potential use for plant improvement facing the challenge of crop adaptation to ongoing climate changes for a sustainable agriculture.

## The Structure and Contents of the Book

The book is divided into 15 chapters each describing different aspects of epigenetics, ranging from fundamental concepts in model plants to applications in plants of agronomic interest. In the first two chapters, authors discuss roles of epigenetic regulation in several aspects of the plant responses to abiotic and biotic stresses and insist on the transient or more sustainable aspect of such regulation. Authors further describe how the memory to both biotic and abiotic stresses can be transfer to the progeny and what are the mechanisms behind this epigenetic inheritance. The third chapter is an update of a chapter published 4 years ago about the inheritance of epialleles. The original thesis—that pure epigenetic variation is rare and often unstable—is still valid, but authors highlight rare situation where pure or facilitated epigenetic alleles can be significant in agronomically interesting traits. In Chapter 4, the book goes on to explain heterosis and the potential involvement of epigenetic mechanisms in this phenomenon in model and crop plants. This chapter also emphasizes opportunities and challenges for epigenetic research to better understand heterosis. Chapter 7 provides a fundamental overview of core histone family and their variants in higher plants. Authors review their evolutionary origin, their diversification, with the emergence of plant-specific histone variants, and their functions regarding all aspects of plant development, focusing especially on agronomically important traits. Chapter 8 discusses the importance of epigenetics in light signaling. Authors highlight that most knowledge were acquired from the model plant Arabidopsis, and because the future of agriculture will most-likely include indoor farming and artificial light, there is a strong need to stimulate more work on plants with commercial value. In chapter 5, 6, and 9, authors focused on agronomically interesting crops. Chapter 5 compiles recent improvement in exploring epigenetic changes in cereal (rice, maize, and wheat) and legumes (cowpea and faba or common bean), as well as in hemp when exposed to abiotic stress and discuss potential roles of epigenetic in controlling the plant stress responses. Chapter 6 describes some epigenetic variations behind the silencing of specific homoeologous genes in polyploid crop species (wheat, cotton). Finally, in chapter 9, readers will learn more about the recent and main findings on tomato epigenetics. In this chapter, the authors also discuss how the combination of epigenetic and genetic natural variation will help to improve tomato breeding in the future. Chapter 10 focuses on CRISPR activation (CRISPRa) technologies for the implementation of targeted modification of epigenetic marks, as an emerging alternative to conventional breeding. Authors emphasize the potential of the epigenome editing technology in improving agronomically important traits such as disease resistance to reduce economic losses. Next, in chapter 11, authors concentrate on the current information on the new research field of epitranscriptomic in Arabidopsis and emphasize how RNA epigenetics may play crucial roles in plant development and acclimation. Chapter 12 and 13 compile and integrate functions of small non-coding RNAs and Polycomb Repressive Complexes in controlling cell plasticity and cell dedifferentiation, as well as zygotic but also somatic embryogenesis. In these two chapters, authors point out that exploring the role of these two epigenetic processes in somatic embryogenesis will be fundamental to successfully achieve massive regeneration of economically interesting crops. Chapter 14 goes into details on the different epigenetic mechanisms involved in germline regulation and male inheritance. These mechanisms are still poorly documented but understanding how epialleles, DNA methylation and other epigenetic mechanism underlying desirable traits are inherited will certainly allow to improve the crop yield and to face the worldwide increasing food need. The final chapter is interested in epigenetics in forest trees. Indeed, besides being involved in regulating developmental processes, epigenetic mechanism can respond to external stimuli, such as long-term cold exposure. Authors also pointed out difficulties of working with trees and future challenges, since the transgenerational inheritance of some epigenetic marks could quickly help to improve tree stress tolerance.

## Conclusion

Epigenetics is a very quickly evolving field of science, but even if a wide expanse of knowledge has been covered, much more remains to be explored. The new edition of the book Epigenetics in Plants of Agronomic Importance brings to the readers the up-to-the-minute fundamental knowledge of epigenetic processes and explicitly states their many interesting and promising applications in agronomically interesting crops. The information brought by this timely new edition should stimulate more work in the field of plant epigenetics and catalyze new discoveries in model plants but also in plants of agronomic interest, as innovative options for breeding applications. Overall it is now urgent to take epigenetics into account in plant breeding strategies to face food security challenges in the context of climate change.

## Author Contributions

DT and AB both read the book and wrote the review.

### Conflict of Interest Statement

The authors declare that the research was conducted in the absence of any commercial or financial relationships that could be construed as a potential conflict of interest.
